# Cholecystocolonic Fistula Associated With Advanced Mirizzi Syndrome Causing Chronic Diarrhea: Successful Treatment Using Cholangioscopy‐Guided Lithotripsy and Surgery

**DOI:** 10.1155/crgm/6770850

**Published:** 2026-07-19

**Authors:** Koichiro Miyagawa, Daiki Uchihara, Shinji Oe, Yasuhisa Mori, Yuichi Honma, Masaru Harada

**Affiliations:** ^1^ The Third Department of Internal Medicine, School of Medicine, University of Occupational and Environmental Health, Kitakyushu, Japan, uoeh-u.ac.jp; ^2^ Department of Surgery, School of Medicine, University of Occupational and Environmental Health, Kitakyushu, Japan, uoeh-u.ac.jp

**Keywords:** cholangioscopy, cholecystocolonic fistula, chronic diarrhea, electrohydraulic lithotripsy, Mirizzi syndrome

## Abstract

**Background:**

Cholecystocolonic fistula (CCF) is a rare complication of advanced Mirizzi syndrome (MS) and often presents with chronic watery diarrhea due to abnormal bile entry into the colon. The optimal role of endoscopic treatment in MS with CCF remains uncertain.

**Case Summary:**

A 63‐year‐old man presented with three months of chronic watery diarrhea. Computed tomography, magnetic resonance imaging, and endoscopic retrograde cholangiopancreatography showed an impacted cystic duct confluence stone, pneumobilia, and contrast passage into the colon, establishing the diagnosis of MS complicated by CCF. A biliary stent improved his symptoms, and peroral cholangioscopy–guided electrohydraulic lithotripsy subsequently enabled complete stone clearance. Cholangioscopy revealed no grossly apparent neoplastic lesions within the gallbladder. The patient subsequently underwent subtotal cholecystectomy with partial colectomy without biliary reconstruction. Histopathology revealed chronic cholecystitis without malignancy, and the postoperative course was uneventful.

**Conclusion:**

A stepwise approach combining cholangioscopy‐guided lithotripsy with targeted surgery can reduce operative complexity and facilitate safe management of advanced MS with CCF.

## 1. Introduction

Mirizzi syndrome (MS) is characterized by chronic gallbladder inflammation caused by an impacted cystic duct stone, occasionally resulting in fistulous communication with adjacent organs. Among these fistulas, cholecystocolonic fistula (CCF) is rare and often manifests as chronic watery diarrhea due to excessive colonic bile acid exposure [1, 2].

Management of MS complicated by CCF is challenging [3]. Severe inflammation and anatomical distortion increase the risk of operative bile duct and vascular injury, while the contribution of endoscopic therapy to surgical simplification remains uncertain.

We present a rare case of advanced MS with CCF successfully managed through a stepwise approach. Cholangioscopy‐guided electrohydraulic lithotripsy (EHL) enabled effective stone clearance and facilitated subsequent safe surgical treatment without the need for biliary reconstruction. This report highlights key diagnostic considerations and suggests that a stepwise treatment strategy may contribute to safer surgical management in complex biliary disease.

This case report was prepared in accordance with the CARE reporting guideline.

## 2. Case Report

A 63‐year‐old man presented with diarrhea persisting for three months. His medical history included obesity (body mass index of 33 kg/m^2^), dyslipidemia, hypertension, and hyperuricemia, for which he was receiving pharmacological treatment. Abnormal liver function tests had been noted during routine health checkups. Given his obesity, increased hepatic echogenicity on abdominal ultrasound, and absence of alcohol consumption, he was diagnosed with metabolic dysfunction–associated steatotic liver disease. Over the past 3 months, he experienced frequent watery diarrhea more than five times daily, occasionally with fecal incontinence, but no abdominal pain, fever, jaundice, pruritus, or pale stools. There was no history of biliary disease, surgery, endoscopic procedures, or trauma. Medications included valsartan, aspirin, ezetimibe, and allopurinol. On examination, he was afebrile and hemodynamically stable. There was no scleral icterus or conjunctival pallor. Abdominal examination showed hyperactive bowel sounds without tenderness. Laboratory tests demonstrated elevated hepatobiliary enzymes: total bilirubin was 0.7 mg/dL, aspartate aminotransferase was 63 U/L, alanine aminotransferase was 78 U/L, alkaline phosphatase was 154 U/L, and gamma‐glutamyl transferase was 91 U/L. Renal function and electrolytes were normal (blood urea nitrogen of 13 mg/dL, creatinine of 0.69 mg/dL, sodium of 143 mmol/L, and potassium of 4.1 mmol/L). C‐reactive protein was slightly elevated (2.42 mg/dL), white blood cell count was 8900/μL, and prothrombin time was slightly prolonged (PT%: 75.7%, international normalized ratio: 1.16). Carbohydrate antigen 19‐9 was normal at 7.4 U/mL. Abdominal ultrasonography was limited due to obesity. Contrast‐enhanced computed tomography (CT) showed indistinct boundaries between the gallbladder and liver, with surrounding hepatic parenchyma of lower density (Figure 1a). Pneumobilia was observed, and the cystic duct appeared disrupted and dilated, losing its normal anatomy (Figure 1b, c). Multiple small hyperdense foci were seen within the biliary tract, though no definitive gallstones were identified at the cystic duct–common bile duct confluence. Thickening of the adjacent colonic wall and an indistinct border between the gallbladder and colon were also noted (Figure 1d). T2‐weighted magnetic resonance imaging (MRI) demonstrated multiple hypointense areas within the gallbladder and a 24‐mm oval‐shaped hypointense lesion at the cystic duct–common bile duct confluence (Figure 2a). Endoscopic retrograde cholangiopancreatography (ERCP) revealed a radiolucent area measuring over 20 mm at the cystic duct confluence, with loss of its normal spiral configuration and luminal dilation. Multiple radiolucent areas were seen within the gallbladder, and contrast medium flowed into the colon (Figure 2b). These findings led to a diagnosis of MS complicated by CCF, corresponding to Csendes Type Va [4]. Gallstone extraction was deferred, and a biliary plastic stent was placed (Figure 2c). Diarrhea improved 2 days after stent placement. One month later, EHL was performed under cholangioscopic guidance for the impacted stone at the confluence of the cystic duct, as described below. After sphincterotomy, the SpyGlass Direct Visualization System (SpyGlass DS II; Boston Scientific, Natick, MA, USA) visualized the stone at the cystic duct confluence (Figure 3a). EHL using the Autolith system (Boston Scientific) fragmented the stone (Figure 3b), and fragments were removed using basket and balloon catheters (Figure 3c). Cholangioscopic inspection of the gallbladder was conducted to confirm the absence of any obvious neoplastic lesions. Colonoscopy revealed a depressed lesion at the hepatic flexure, suspected to be the fistula (Figure 3d, e). No tumor was observed, and marking clips were placed for intraoperative identification. Bile leakage was absent, possibly due to stone removal. One month later, prior to surgery, repeat ERCP confirmed no residual stones, and an endoscopic nasobiliary drainage tube was placed (Figure 3f). The patient underwent open subtotal cholecystectomy with partial colectomy. The surgical specimen demonstrated a fistulous tract from colonic mucosa to gallbladder mucosa (Figure 4a). Histopathology revealed chronic cholecystitis without malignancy, including the fistula site (Figure 4b, c). The patient was discharged on postoperative day seven and remains symptom‐free, with no evidence of recurrent biliary disease or malignancy during 33 months of follow‐up based on serial laboratory testing and surveillance contrast‐enhanced CT.

**FIGURE 1:**
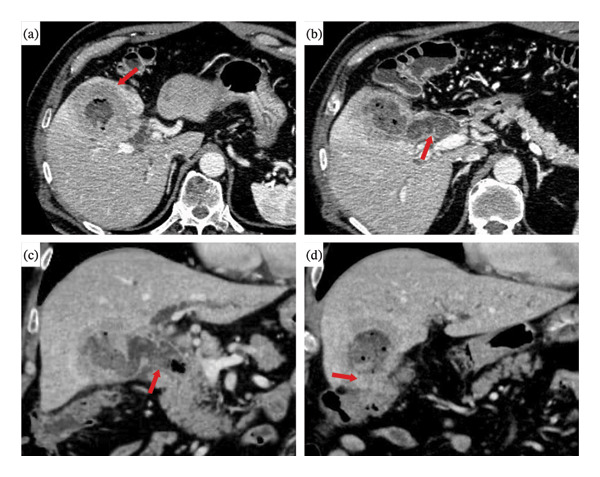
Contrast‐enhanced computed tomography findings on admission ((a, b) axial images; (c, d) coronal images). (a) Indistinct border between the gallbladder and liver, with low attenuation in adjacent hepatic parenchyma (red arrow). (b, c) Pneumobilia and disrupted, dilated cystic duct with loss of normal configuration (red arrow). (d) Thickened colonic wall adjacent to the gallbladder and unclear gallbladder–colon border (red arrow).

**FIGURE 2:**
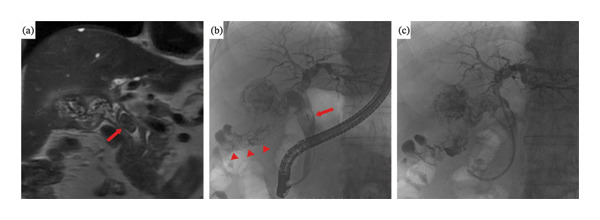
Magnetic resonance imaging (MRI) and endoscopic retrograde cholangiopancreatography (ERCP) findings supporting the diagnosis of Mirizzi syndrome with cholecystocolonic fistula. (a) T2‐weighted MRI showing multiple hypointense areas in the gallbladder and a 24‐mm hypointense lesion at the confluence of the cystic duct and common bile duct (red arrow). (b) ERCP demonstrating a radiolucent area (red arrow) at the cystic duct junction with ductal dilation and disrupted anatomy; contrast medium flows into the colon (red arrow heads). (c) Biliary stent placed without stone extraction.

**FIGURE 3:**
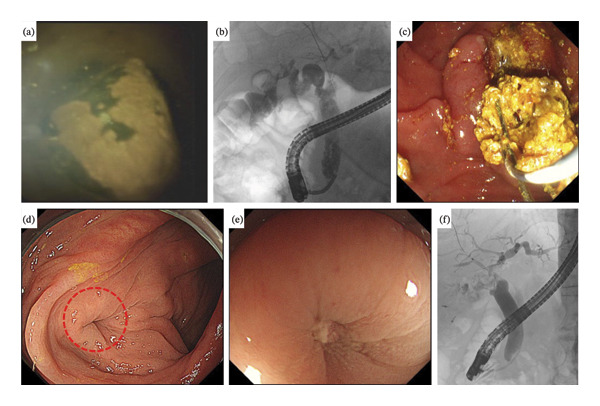
Endoscopic treatment of gallstone and colonoscopic evaluation of the cholecystocolonic fistula. (a) Peroral cholangioscopy visualizing a stone at the confluence of the cystic duct. (b) Post‐lithotripsy cholangiography showing multiple fragments. (c) Stone fragments removed via basket catheter. (d) Colonoscopy revealing a fistula as a depression at the hepatic flexure (red dotted circle). (e) Close‐up image of the fistula site. (f) Follow‐up endoscopic retrograde cholangiopancreatography confirming no residual stones.

**FIGURE 4:**
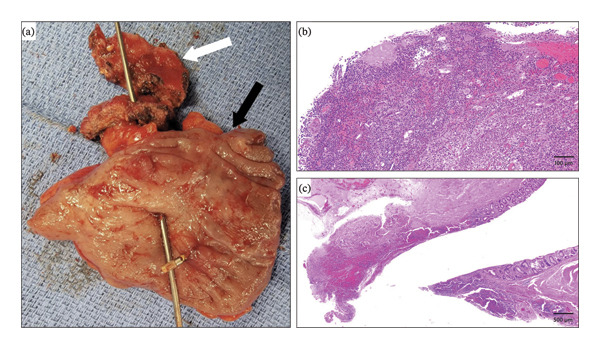
Pathological examination of the surgical specimen. (a) The white arrow indicates the gallbladder, and the black arrow indicates the colon. A probe is inserted through the fistula. A marking clip is visible on the colon. (b) Histological image of the resected gallbladder. The mucosal surface shows proliferative granulation tissue with multinucleated giant cells and dense infiltration of chronic inflammatory cells. No evidence of significant epithelial atypia is identified. (c) Histological image of the fistula site. Mild chronic inflammatory cell infiltration, edema, and hemorrhage are observed throughout the wall, along with mucosal disruption. No evidence of epithelial atypia is identified.

## 3. Discussion

Biliary fistula complicated with various biliary diseases is an abnormal communication between the biliary tract and other organs, classified as external or internal. Internal biliary fistulas form between the gallbladder and the gastrointestinal tract, most commonly the duodenum, followed by the colon [1]. Chronic cholecystitis caused by cystic duct obstruction promotes adhesion between the gallbladder and adjacent organs, leading to necrosis and fistula formation. Besides gallstones, causes include gallbladder carcinoma, trauma, inflammatory bowel disease, and colorectal cancer [5]. Savvidou et al. proposed a triad of pneumobilia, chronic diarrhea, and vitamin K malabsorption as characteristic of CCF [6]. However, this has not been empirically validated, and its prevalence among CCF patients remains unclear. Chronic diarrhea is considered the most common symptom, reported in approximately 70% of cases [2]. Bile acids entering the colon increase motility, mucosal permeability, and chloride secretion, causing diarrhea [7]. Normally, bile acids are reabsorbed in the terminal ileum via the ileal bile acid transporter (IBAT), activating farnesoid X receptor (FXR) and inducing fibroblast growth factor 19 (FGF19), which suppresses hepatic bile acid synthesis [8]. Disruption of the FXR‐FGF19–mediated negative feedback pathway has been proposed as a potential mechanism of diarrhea in patients with CCF. Although this mechanism may explain the symptoms observed in our patient, bile acid and FGF19 levels were not evaluated. Therefore, the involvement of the FXR‐FGF19 pathway in this patient’s diarrhea remains speculative.

MS is characterized by chronic gallbladder inflammation due to impacted gallstones, leading to adhesion with the common bile duct, biliary stricture, and in some cases, cholecystobiliary fistula formation. However, its pathophysiology extends beyond local anatomical disruption. Persistent inflammation in Calot’s triangle may involve adjacent organs, resulting in complex fistulous connections [9]. Beltran et al. found that among 5783 cholecystectomies, 105 cases (1.8%) had CCF, 94 (89.5%) of which were associated with MS [4], suggesting that the presence of CCF should raise suspicion for concurrent MS. However, the preoperative diagnosis of MS can be challenging. Seah et al. reported that only 75% of MS cases (48 out of 64) were correctly diagnosed prior to surgery [10]. Among imaging modalities, ultrasonography is useful for detecting gallstones and biliary dilation but has limited specificity for MS, with a diagnostic accuracy ranging from 10% to 50% [10–12]. CT helps differentiate from malignancy, but its diagnostic yield remains modest when used in isolation, with values of approximately 40% [10, 13]. MRCP enables noninvasive assessment of biliary anatomy and demonstrates high diagnostic accuracy, reported to be 78%–89% [10, 14]. Endoscopic ultrasonography provides detailed evaluation of the gallbladder and adjacent structures, with a sensitivity of 63% [15]. ERCP is also valuable, with reported diagnostic accuracy ranging from 55% to 90% [16]. These findings underscore the clinical importance of a comprehensive multimodal imaging approach to assess the potential presence of MS when CCF is identified.

Surgical intervention remains the mainstay of treatment for MS, with the approach guided by the Csendes classification [4]. The present case was classified as Csendes Type Va because a CCF was present without gallstone ileus. Type I and select II/III cases allow subtotal or total cholecystectomy [17]. Advanced Type II/III or Type IV, characterized by severe adhesions in Calot’s triangle, may require biliary reconstruction [16]. Type V, complicated by CCF, typically involves fistula takedown, along with either total or subtotal cholecystectomy [9, 16]. In the present case, severe inflammation and adhesions in Calot’s triangle were anticipated, presenting a considerable risk of right hepatic artery and bile duct injury during cholecystectomy [18]. Consequently, bile duct resection and biliary reconstruction would typically be required. However, considering the high risk of complications, we chose stepwise approach using cholangioscopy‐guided EHL followed by targeted surgery to minimize surgical manipulation in Calot’s triangle.

A recent report described a less invasive strategy combining EHL for stone fragmentation with over‐the‐scope clip closure of CCF [19]. However, gallbladder carcinoma has been reported in up to 5.3% of MS cases and is particularly associated with advanced stages of MS, especially those complicated by CCF, warranting careful consideration [16, 20]. Therefore, in cases where cholecystectomy is not performed, the absence of histopathological evaluation necessitates prudent patient selection and close postoperative surveillance. In our case, preoperative cholangioscopy enabled direct visualization of the gallbladder, revealing no grossly apparent neoplastic lesions. Subsequent subtotal cholecystectomy confirmed the absence of malignancy. During 33 months of follow‐up, including serial laboratory testing and surveillance contrast‐enhanced CT, the patient has remained asymptomatic, with no evidence of recurrent biliary disease or malignancy. Nevertheless, given the reported association between advanced MS and gallbladder carcinoma, continued long‐term surveillance is warranted to assess for the possible development of malignancy in the remnant gallbladder.

In conclusion, CCF should be considered in patients with unexplained chronic diarrhea. Its strong association with advanced MS warrants multimodal imaging for diagnosis. Stepwise management combining cholangioscopy‐guided lithotripsy and targeted surgery provides a safe, effective, and less invasive treatment strategy. This case emphasizes the importance of careful preoperative assessment and individualized surgical planning in complex biliary disease.

## Author Contributions

All authors contributed equally to manuscript preparation, imaging review, and manuscript revision.

## Funding

No funding was received for this publication.

## Ethics Statement

Institutional Review Board approval was waived because this study is a single case report.

## Consent

Written informed consent was obtained from the patient for publication of this case report and accompanying images.

## Conflicts of Interest

The authors declare no conflicts of interest.

## Supporting Information

Additional supporting information can be found online in the Supporting Information section.

## Supporting information


**Supporting Information** CARE‐checklist‐English‐2013.
