# Supplementary material for: Cholecystocolonic Fistula Associated With Advanced Mirizzi Syndrome Causing Chronic Diarrhea: Successful Treatment Using Cholangioscopy‐Guided Lithotripsy and Surgery
Source: Case Rep Gastrointest Med. 2026 Jul 19;2026:6770850. doi: 10.1155/crgm/6770850 (PMC13382152; doi:10.1155/crgm/6770850)
Supplement: Supplementary file 1 — Supporting Information CARE‐checklist‐English‐2013. [file CRGM-2026-6770850-s001.pdf]

CARE case report guidelines

CARE Checklist of information to include when writing a case report

(cc) BY-NC-ND

Topic	Item	Checklist item description	Reported on Line
**Title**	1	The diagnosis or intervention of primary focus followed by the words "case report"	Title page
**Key Words**	2	2 to 5 key words that identify diagnoses or interventions in this case report, including "case report"	Page 2, lines 19-20
**Abstract (no references)**	3a	Introduction: What is unique about this case and what does it add to the scientific literature?	Page 2, lines 2-5
	3b	Main symptoms and/or important clinical findings	Page 2, lines 6-7
	3c	The main diagnoses, therapeutic interventions, and outcomes	Page 2, lines 7-14
	3d	Conclusion—What is the main "take-away" lesson(s) from this case?	Page 2, lines 15-17
**Introduction**	4	One or two paragraphs summarizing why this case is unique (**may include references**)	Page 3, lines 1-15
**Patient Information**	5a	De-identified patient specific information.	Page 3, line 17
	5b	Primary concerns and symptoms of the patient.	Page 3, lines 17 and 23 & Page 4, lines 1-2
	5c	Medical, family, and psycho-social history including relevant genetic information	Page 3, lines 17-23
	5d	Relevant past interventions with outcomes	No relevant past interventions
**Clinical Findings**	6	Describe significant physical examination (PE) and important clinical findings.	Page 4, lines 4-6
**Timeline**	7	Historical and current information from this episode of care organized as a timeline	Additional file for review not for publication
**Diagnostic Assessment**	8a	Diagnostic testing (such as PE, laboratory testing, imaging, surveys).	Page 4, line 6 - Page 5, line 4
	8b	Diagnostic challenges (such as access to testing, financial, or cultural)	Not applicable
	8c	Diagnosis (including other diagnoses considered)	Page 5, lines 12-16
	8d	Prognosis (such as staging in oncology) where applicable	Not applicable
**Therapeutic Intervention**	9a	Types of therapeutic intervention (such as pharmacologic, surgical, preventive, self-care)	Page 5 lines 5-12 & Page 5 lines 17-19
	9b	Administration of therapeutic intervention (such as dosage, strength, duration)	Not applicable
	9c	Changes in therapeutic intervention (with rationale)	Not applicable
**Follow-up and Outcomes**	10a	Clinician and patient-assessed outcomes (if available)	Page 5 line 22 - Page 6 line 2
	10b	Important follow-up diagnostic and other test results	Page 5 line 22 - Page 6 line 2
	10c	Intervention adherence and tolerability (How was this assessed?)	Not applicable
	10d	Adverse and unanticipated events	None
**Discussion**	11a	A scientific discussion of the strengths AND limitations associated with this case report	Page 8, lines 3-9 and lines 21-23
	11b	Discussion of the relevant medical literature **with references.**	Checked
	11c	The scientific rationale for any conclusions (including assessment of possible causes)	Page 6, lines 14-23
	11d	The primary "take-away" lessons of this case report (without references) in a one paragraph conclusion	Page 9, lines 1-6
**Patient Perspective**	12	The patient should share their perspective in one to two paragraphs on the treatment(s) they received	Page 5 line 22 - Page 6 line 2
**Informed Consent**	13	Did the patient give informed consent? Please provide if requested	**Yes** ☑ **No** ☐
